# Long-Term Survivors in a Cohort of People Living with HIV Diagnosed between 1985 and 1994: Predictive Factors Associated with More Than 25 Years of Survival

**DOI:** 10.3390/idr15010008

**Published:** 2023-01-20

**Authors:** Federica Cosentino, Andrea Marino, Laura Anile, Vittoria Moscatt, Maria Gussio, Vincenzo Boscia, Roberto Bruno, Giuseppe Nunnari, Alfredo Pulvirenti, Grete Francesca Privitera, Bruno Santi Cacopardo, Manuela Ceccarelli, Benedetto Maurizio Celesia

**Affiliations:** 1 Unit of Infectious Diseases, Department of Clinical and Experimental Medicine, University of Messina, 98124 Messina, Italy; 2 Unit of Infectious Diseases, “Garibaldi” Hospital, 95122 Catania, Italy; 3 Unit of Infectious Diseases, Department of Biomedical and Biotechnological Sciences, University of Catania, 95123 Catania, Italy; 4 Unit of Pediatrics, Department of Medical and Surgical Sciences, “Alma Mater” University of Bologna, 40126 Bologna, Italy; 5 Unit of Infectious Diseases, Department of Clinical and Experimental Medicine, University of Catania, 95122 Catania, Italy; 6 Unit of Informatics, Department of Clinical and Experimental Medicine, University of Catania, 95123 Catania, Italy; 7 Unit of Infectious Diseases, Department of Biomedical, Dental, Morphological and Functional Imaging Sciences, University of Messina, 98124 Messina, Italy

**Keywords:** HIV epidemiology, long-term survivors, HAART

## Abstract

Although the mortality rate among individuals diagnosed during the pre-Highly Active Antiretroviral Treatment era has been substantial, a considerable number of them survived. Our study aimed to evaluate the prevalence of HIV long-term survivors in a cohort of People Living with HIV diagnosed between 1985 and 1994 and to speculate about potential predictive factors associated to long survival. This is a retrospective single-center study. Subjects surviving more than 300 months (25 years) from HIV diagnosis were defined as Long Term Survivors. Overall, 210 subjects were enrolled. More than 75.24% of the included people living with HIV were males, with a median age of 28 years (IQR 25–34). The prevalent risk factors for HIV infection were injection drug use (47.62%), followed by unprotected sex among heterosexual individuals (23.81%). Ninety-three individuals (44.29%) could be defined as LTS with a median (IQR) survival of 333 (312–377) months. A hazard ratio of 12.45 (95% CI 7.91–19.59) was found between individuals who were exposed to Highly Active AntiRetroviral Treatment (HAART) and individuals who were not, with the latter being at greater risk of death. The availability and accessibility of effective antiretroviral therapy for people living with HIV remain the cornerstone of survival.

## 1. Introduction

Since the beginning of the epidemic, the human immunodeficiency virus (HIV) radically changed lives of millions of women and men in the world. The World Health Organization (WHO) estimated that in 2021, 38.4 million people were living with HIV infection, and 650,000 people died because of HIV [1]. The burden of the epidemic continues to vary considerably between countries and regions [1,2]. HIV infection is still a serious public health challenge [3]. Although the mortality rate among People Living with HIV (PLWH) diagnosed during the pre-Highly Active Antiretroviral Therapy (HAART) era was substantial, a considerable number of those individuals are still alive [4,5]. The introduction of HAART in 1996 led to a decreased rate of acquired immunodeficiency syndrome (AIDS)-related morbidity and mortality [4,6,7]. Nowadays, the population of Long-Term Survivors (LTS, that is, PLWH who survived for more than 25 years) is very large [8,9,10]. The first generation of LTS includes subjects who acquired the infection between the 1980s and 1990s, before the advent of HAART, and who are therefore defined as pre-HAART LTS to distinguish them from post-HAART LTS [11]. 

Many factors have been associated with survival in PLWH. Adherence to an effective antiretroviral (ARV) treatment and the degree of immunodeficiency at the time of combined antiretroviral therapy (cART) initiation seem to be the most important ones [12,13]. CD4+ count has been proposed as a potential determinant of survival in combination with other clinical data such as sex, race, presence of comorbidities, clinical acquired immunodeficiency syndrome (AIDS), and age at HAART initiation [14]. Furthermore, some studies have shown that HIV/HCV coinfection is related to a rapid progression of liver damage, with an increased probability of earlier development of end-stage liver disease [7,15,16]. 

Understanding the factors that contribute to long-term survival is crucial for providing further insight into the natural history and pathophysiology of HIV infection, for comparing the mortality rates in PLWH with those of the general population, and for developing longer-term survival models. The aim of this study is to evaluate the prevalence of LTS in a cohort of PLWH diagnosed between 1985 and 1994 and to speculate about potential predictive factors associated with long survival. Therefore, we evaluated epidemiological and clinical parameters recorded at the time of diagnosis.

## 2. Materials and Methods

We conducted a retrospective observational monocentric study. All subjects with a diagnosis of HIV infection confirmed with a Western Blot assay between 1 May 1985 and 31 December 1994 in follow-up at the Infectious Diseases outpatient clinic of the Garibaldi Nesima Hospital in Catania, Italy, were considered eligible for the study. This research was conducted according to the Declaration of Helsinki. Subjects signed a general written informed consent to the use of their data for research purposes upon the admission. The data were collected by a member of the medical team that treated each individual, and these data have been codified and anonymized before their statistical analysis. For the retrospective design of the study, based on a database enquiry and medical records consultation, we did not request ethical committee approval.

### 2.1. Data Collection

Epidemiological and clinical data registered at the time of HIV diagnosis were collected from medical records using an electronic worksheet. The clinical and laboratory parameters recorded at diagnosis and at the time of the last visit to the clinic were considered for the purposes of the study. Specifically, we obtained the following information:

- Epidemiological characteristics: sex, age, HIV exposure (IVDUs, MSMs, heterosexual transmission, transfusions).
- Clinical parameters: date of HIV diagnosis; baseline CD4+ T-cells count (cells/µL); CD8+ T-cells count (cells/µL); CD4+/CD8+ ratio; CD4+ nadir; HIV-RNA viral load (copies/mL), if available; HBsAg and HCV antibody test results; the first and the last recorded laboratory tests, including hemoglobin (g/dL), platelets count (×10^3^/µL), creatinine (mg/dL), aspartate aminotransferase (UI/L), alanine aminotransferase (UI/L); date of most recent visit; antiretroviral therapy before the first cART; data of starting cART; AIDS-defining illnesses; AIDS presenter; date and cause of death.

### 2.2. Measurement of T-Cell Lymphocytes Subsets and HIV-RNA Plasma Viral Load

CD4^+^ T-cell count, CD8^+^ T-cell count, and CD4^+^/CD8^+^ ratio were measured by cytofluorimetry with BD FACS^®^ Canto II (BD Biosciences, Becton Dickinson and Co, Franklin Lakes, NJ, USA), using BD Multitest™ (BD Biosciences, Becton Dickinson and Co, Franklin Lakes, NJ, USA) and four-color direct immunofluorescence reagent according to the manufacturer’s instructions. The BD Multitest™ includes CD3 fluorescein isothiocyanate (FITC), CD8 phycoerythrin (PE), CD45 peridinin chlorophyll protein (PerCP), CD4 allophycocyanin (APC). 

Plasma viral load was measured in 400 µL of plasma with Cobas^®^ HIV-1 (Roche, Basel, Switzerland) according to the manufacturer’s instructions. The lower limit of detection is 20 cps/mL; the higher limit of detection is 10^7^ cps/mL.

The effectiveness of ART was evaluated by the achievement of an undetectable plasma HIV-RNA viral load (<50 copies/mL) since its measurement became available. Longitudinal observation was stopped on 31 December 2019, that is, before the beginning of the SARS-CoV-2 pandemic. Subjects surviving more than 300 months from HIV diagnosis were defined LTS.

### 2.3. Inclusion Criteria

- All subjects with an ELISA antibody test positive for HIV, as confirmed with a Western Blot assay between 1 May 1985 and 31 December 1994.
- Age > 18 years.

### 2.4. Exclusion Criteria

- PLWH who were lost to follow-up or transferred to another ID center before completing the entire observation period (31 December 2019).
- Individuals who received the first diagnosis elsewhere.

### 2.5. Statistical Analysis

Continuous variables have been presented as median and interquartile range (IQR), whereas the categorical variables have been expressed with frequencies and percentages. In the evaluation of the parameters that were stated as not normally distributed thanks to the Shapiro–Wilk test of normality, the non-parametric Wilcoxon rank-sum test was applied. The Kaplan–Meier estimator was used for survival analyses building survival curves. Hazard Ratio was estimated through Cox proportional hazards regression model. To understand the relation between variables and survival, we used linear regression. Statistical analysis was performed with jamovi 2.0.0.0 for MacOS and Graphpad Prism 9.4.1 (Graphpad software, San Diego, CA, USA).

## 3. Results

### 3.1. Characteristics of the Population

A total of 224 subjects were initially identified; of these, 14 individuals were excluded because they were lost to follow-up soon after the diagnosis. Overall, 210 PLWH, in follow-up at the Infectious Disease Department at Garibaldi Nesima Hospital in Catania, were enrolled. Characteristics of the cohort are summarized in Table 1. The population was predominantly male [n = 158 (75.24%)], and the median age was 28 years (IQR 25–34). The prevalent risk factors for HIV infection were injection drug use [IDU, n = 100 (47.62%)], followed by unprotected sex among heterosexual people [n = 50 (23.81%)], and men who have sex with men [MSM, n = 42 (20%)] individuals. Blood transfusion accounted for 6.19%. A total of 69 (32.86%) individuals were AIDS presenters at diagnosis, and 116 (55.24%) had any AIDS diagnosis (ADI) during the period included in the study. One-hundred and one (48.10%) individuals were found to be positive for anti-HCV antibodies. Importantly, 77.2% of these HCV+ PLWH were IDUs. The median CD4+ cell count at diagnosis was 234 cells/μL (IQR 54–439), and the median CD4+ nadir was 35 cells/μL (IQR 14–94), although these data were known for only 129 subjects. The number of PLWH who were treated with pre-HAART single-drug or dual-drug ARV therapy was 125 (59.52%), whereas 120 (57.14%) PLWH received a HAART regimen.

**Figure 1:**
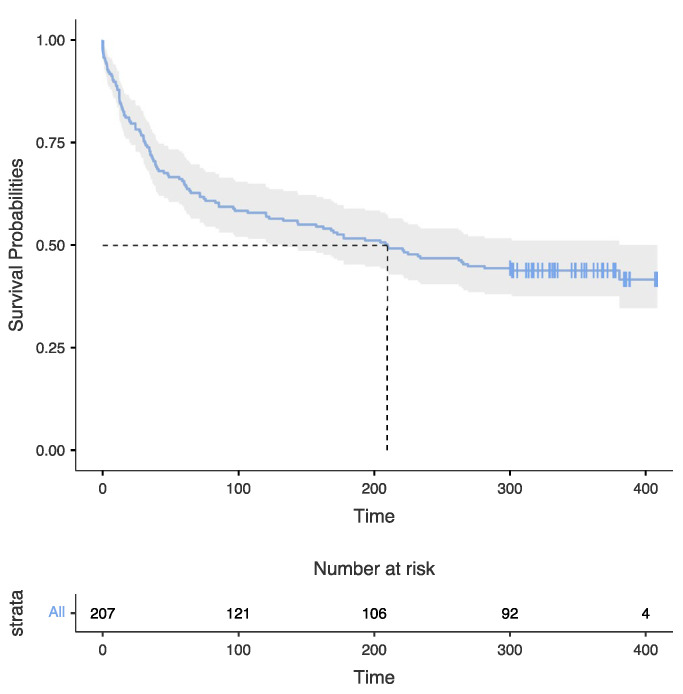
Overall survival among HIV population. This figure shows the overall survival of the cohort of People Living with HIV (PLWH) diagnosed between 1985–1994 in follow-up at the HIV outpatient clinic of the Unit of Infectious Diseases of the “Garibaldi” Hospital in Catania, Italy. At the end of the period of observation, 42.9% of the cohort was still alive.

Overall survival is shown in Figure 1.

Median time of survival for this cohort was 209 months (IQR 31–328). At the end of the period of observation, 90 individuals (42.9%) were still alive. The main cause of death was an AIDS-related event (Table 2). We divided the cohort into two subgroups, according to their time of survival, either higher or lower than 300 months. Ninety-three individuals (44.29%) could be defined as LTS with a median survival of 333 months (IQR 312–377); 116 (55.24%) subjects died before 25 years (non-long-term survivors—NLTS) with a median survival of 35 months (IQR 12–117).

There is a statistically significant difference in terms of survival between those PLWH who had AIDS at diagnosis and those who were AIDS-free (*p* = 0.034). This difference is even more significant when comparing NLTS and LTS: almost none of the LTS individuals was an AIDS case at diagnosis (*p* < 0.001) (Table 3).

### 3.2. The “HAART Effect”

**Figure 2:**
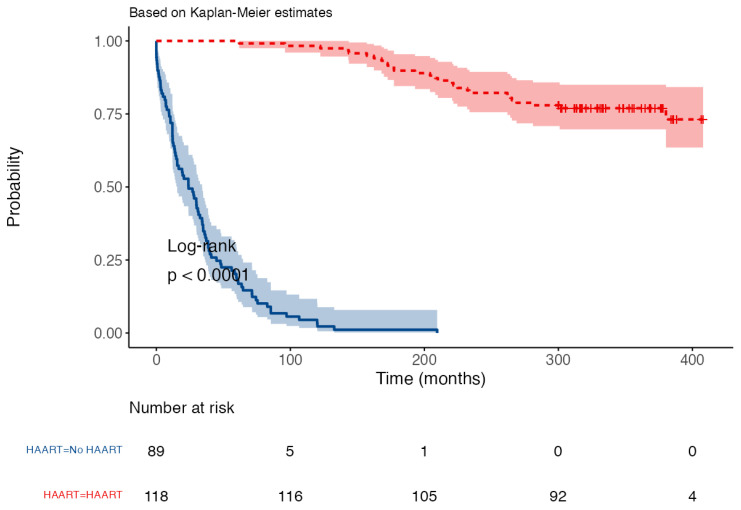
Probability of survival stratified by access to HAART treatment. This figure shows the difference in survival time between individuals who had access to HAART (red line) vs. individuals who did not receive HAART (blue line). Hazard ratio (log-rank) was 12.45 (95% CI 7.91–19.59, *p* < 0.001), with no-HAART subjects being at higher risk of death than people who took HAART. Abbreviations: HAART—highly active antiretroviral treatment.

Overall, 120 PLWH in this cohort were on HAART treatment (57.1%), whereas 90 were not (42.9%). Therefore, we decided to compare overall survival in patients not undergoing HAART treatment vs. patients who took HAART. Hazard ratio, measured with a log-rank method, was 12.45 (95% CI 7.91–19.59), with individuals who were not exposed to HAART being at greater risk of death. We also discovered a statistically significant difference (*p* < 0.001) between the respective median time of survival. In fact, median time of survival in HAART-treated individuals was 316 months (IQR 300–369), whereas median time of survival in no-HAART subjects was 24 months (IQR 9–46). Figure 2 shows the difference in survival times created by the introduction of HAART.

This difference was so significant that we decided to explore the other factors considering the HAART group separately. Moreover, all the LTS patients took HAART; therefore, comparing them with those who did not take HAART would have introduced a bias.

### 3.3. People Living with HIV Who Had Access to HAART

All the 93 LTS underwent HAART, whereas only 27 NLTS (23.1%) had access to HAART. We compared the effects of other factors on differences of survival of these sub-cohorts. Moreover, only two PLWH received HAART after AIDS diagnosis, one LTS and one NLTS.

Firstly, we chose to stratify the whole subgroup of PLWH having access to HAART by CD4+ T-cell count at diagnosis. There is no statistically significant difference between having a CD4+ T-cell count at diagnosis higher than 500 cell/µL, between 200–500 cells/µL, or lower than 200 cells/µL (*p* = 0.154) in terms of survival. However, when we stratified PLWH by mode of transmission, it came out that having a CD4+ T-cell count < 200 cells/µL was significantly associated with a shorter survival (*p* < 0.001). We also stratified both LTS- and NLTS-PLWH by CDC ’93 CD4+ T-cell category, and we did not find any statistically significant difference between survival rate in these sub-groups (*p* = 0.062 and *p* = 0.129, respectively). 

**Figure 3:**
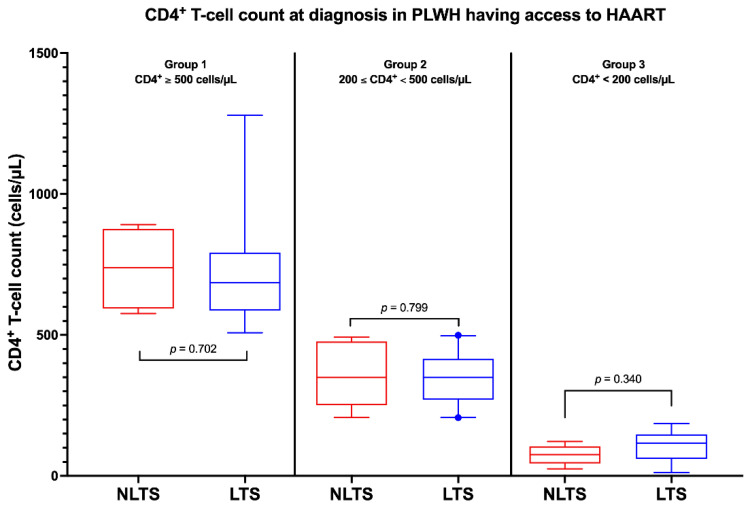
CD4+ T-cell count at diagnosis in People Living with HIV (PLWH) having access to Highly Active Anti-Retroviral Treatment (HAART). This figure shows that in PLWH who had access to HAART, CD4+ T-cell count was not significantly different between non-long-term survivors (red) and long-term survivors (blue), even when stratifying by CDC ’93 category. Whiskers represent the 95% CI. Abbreviations: PLWH—people living with HIV; HAART—highly active antiretroviral therapy; NLTS—non-long-term survivors; LTS—long-term survivors.

We compared median time of survival of different CDC ’93 subgroups of LTS-PLWH and NLTS-PLWH, and we did not find any statistically significant difference (*p* = 0.437 and *p* = 0.060, respectively), although a certain trend towards shorter survival can be seen, especially in NLTS-PLWH with lower CD4+ T-cell counts at diagnosis (Supplemental Figure S1). Moreover, CD4+ T-cell count at diagnosis is not significantly different in NLTS-PLWH and LTS-PLWH who underwent HAART (*p* = 0.351), even when stratifying by CDC ’93 category (Figure 3). These results show that CD4+ T-cell count at diagnosis cannot be considered a predictor of survival in PLWH who had access to HAART.

**Figure 4:**
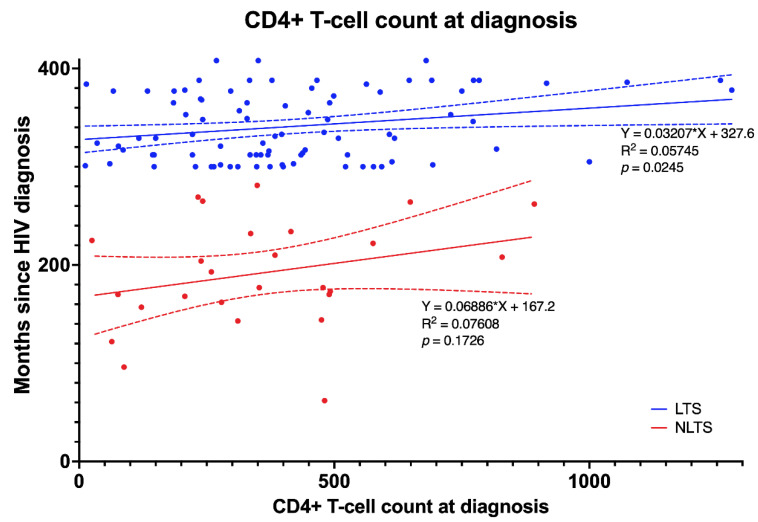
CD4+ T-cell count at diagnosis influences survival time in LTS- but not in NLTS-PLWH on HAART. This figure shows that in LTS-PLWH (blue lines and dots), a statistically significant relationship exists between CD4+ T-cell count at diagnosis and time of survival. Abbreviations: LTS—long-term survivors; NLTS—non-long-term survivors; PLWH—people living with HIV; HAART—highly active antiretroviral therapy.

Although CD4+ T-cell count at diagnosis does not influence the survival rate, linear regression shows that it can influence survival time in LTS but not in NLTS on HAART (Figure 4).

**Figure 5:**
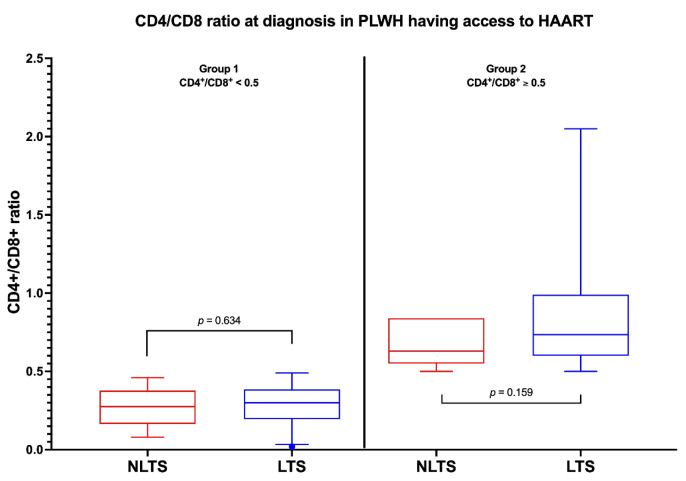
CD4+/CD8+ ratio at diagnosis in PLWH who had access to HAART. This figure shows that at diagnosis, there was no statistically significant difference between NLTS-PLWH (red) and LTS-PLWH (blue) in terms of CD4/CD8 ratio when stratifying by ratio higher or lower than 0.5. Abbreviations: PLWH—people living with HIV; HAART—highly active antiretroviral treatment; NLTS—non-long-term survivors; LTS—long-term survivors.

We then studied the differences in terms of CD4+/CD8+ ratio between LTS- and NLTS-PLWH. We did not find any differences between the median CD4+/CD8+ ratio in LTS and NLTS-PLWH (*p* = 0.206). The difference was not statistically significant, even when stratifying for a CD4+/CD8+ ratio higher (*p* = 0.159) or lower than 0.5 (*p* = 0.634) (Figure 5).

**Figure 6:**
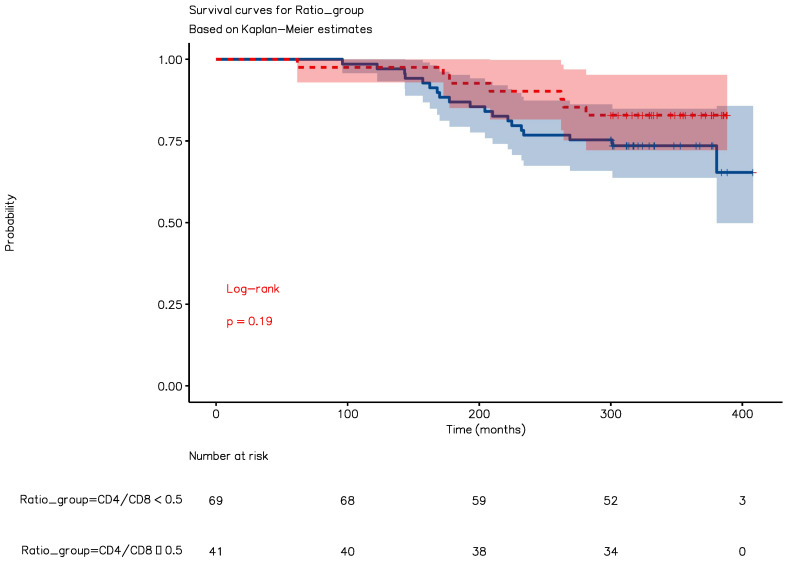
CD4+/CD8+ ratio at diagnosis is not a good predictor of survival. This figure shows that there is no statistically significant difference in terms of survival between those who were diagnosed with a CD4+/CD8+ ratio lower than 0.5 (blue) and those who received their diagnosis when the CD4+/CD8+ ratio was higher than 0.5 (red). All the PLWH included in this analysis received HAART.

Figure 6 shows that there is no statistically significant difference between having a CD4+/CD8+ ratio at diagnosis higher or lower than 0.5 (*p* = 0.19) in terms of survival. Taken together, these data show that CD4+/CD8+ ratio at diagnosis is not a good predictor of long-term survival in patients taking HAART.

We also evaluated the effect on survival time of antiretroviral treatment before HAART (*p* = 0.59), of having HCV co-infection (*p* = 0.27), and of having HBV co-infection (*p* = 0.84). Although having AIDS at diagnosis was a statistically significant predictor of mortality in the whole cohort, this significance was not confirmed when only PLWH having access to HAART were considered (*p* = 0.79).

**Figure 7:**
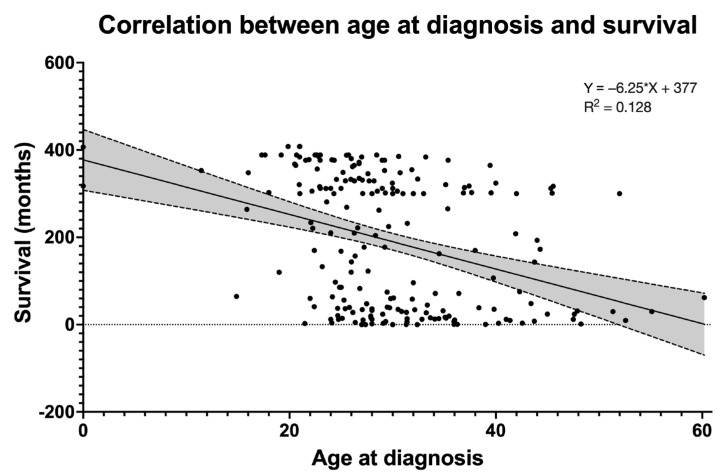
Age at diagnosis influences time of survival. This figure shows that a linear correlation might exist between age at diagnosis and time of survival, although this correlation is weak (R squared 0.128).

**Figure 8:**
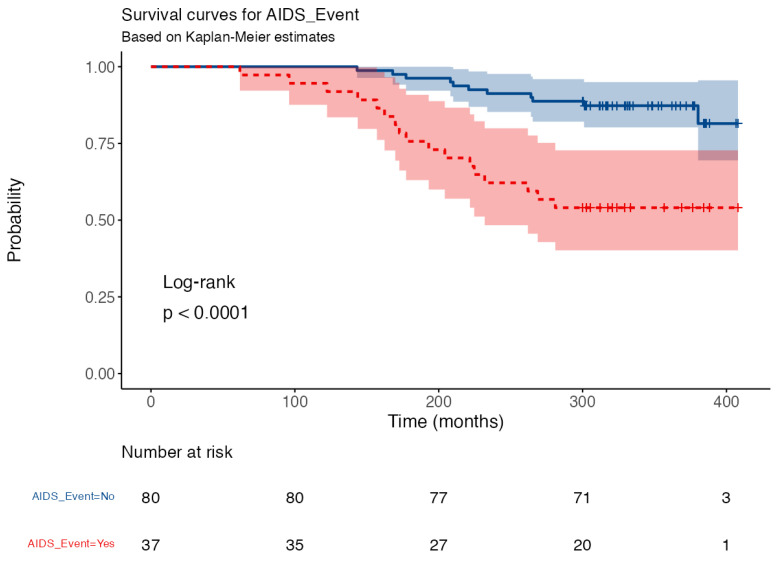
AIDS-related events influence the survival rate in PLWH who had access to HAART. This figure shows that in PLWH who were taking HAART, the appearance of an AIDS-related event is a negative prognostic factor for survival. Hazard ratio of those who had an AIDS-related disease was 4.329 (95% CI 1.866–10.040) compared with those who did not have an AIDS-related event, meaning that they had a 4-fold higher risk of dying. Abbreviations: AIDS—acquired immunodeficiency syndrome; PLWH—people living with HIV; HAART—highly active antiretroviral treatment.

However, age at diagnosis influences time of survival (*p* < 0.001, Figure 7) and showing an AIDS-related disease was found to be a prognostic negative factor for survival. In fact, among those PLWH who had access to HAART, the patients who had an AIDS-related event had a statistically significant lower survival rate (log-rank HR 4.329, 95% CI 1.866–10.040, *p* < 0.001) (Figure 8). These data show that an AIDS-related event is a predictor of survival in patients undergoing HAART. Stratifying by mode of transmission of the infection, AIDS events are significantly more frequent in PLWH who acquired the infection through unprotected homosexual sex than both IDUs (*p* < 0.001) and those who acquired the infection through unprotected heterosexual sex (*p* = 0.027).

## 4. Discussion

Since the beginning of the epidemic, around 84.2 million people worldwide have been diagnosed with HIV, and 40.1 million of them are estimated to have died from AIDS-related illnesses [1]. The introduction of antiretroviral therapy has dramatically changed the prognosis of HIV disease by reducing HIV-related mortality [3]. Many studies have systematically evaluated the nature of relative longevity of PLWH [17,18]. Our retrospective observational study provides a complete description of the demographic, clinical characteristics, and laboratory findings in a population of 210 PLWH recruited in our ID outpatient clinic and aims to identify those factors associated with survival. We evaluated possible determinants of disease progression. 

Our analysis showed a robust association between receiving a cART and survival. This is not surprising given that studies suggest that early effective antiretroviral therapy delayed death and transformed HIV infection from being a fatal disease into a chronic condition. Many studies have found that AIDS-defining illnesses as the cause of death are declining dramatically after HAART introduction [19]. Our analysis strongly shows how use of HAART is protective for survival in PLWH. About 83% of this cohort died of an AIDS-defining condition. Other major causes of death included liver-related causes, non-AIDS malignancy, cardiovascular diseases, and substance abuse. Because all the long-term survivors included in this study had access to HAART, we chose to compare only LTS with NLTS who took HAART.

In our cohort, the main risk factor for HIV infection was intravenous drug use (47.62%). These data are consistent with previous studies [20]. IDUs have been the group at highest risk of new HIV infection in the 90′s in Italy, whereas sexual transmission currently represents the most common route of transmission among MSM and heterosexual people. In our study cohort, heterosexual transmission accounted for 23.81%, whereas MSM represented 20%. PLWH who had a history of blood transfusions as a risk factor were 6.19%. The introduction of blood-screening for HBV, HIV (from 1985), and HCV in the early 1990s strongly decreased transmission of these infections from blood transfusions. HIV/HCV coinfection has been identified in 48.10% of this cohort. 

Many studies have demonstrated that HIV/HCV coinfection is related to a rapid progression of the liver damage showing the benefit of the HCV eradicative therapy for the health of PLWH; however, we did not find any association between HCV and survival rate [7]. We collected data about only HCV serology; therefore, it is not possible to evaluate how spontaneous clearance of the virus might have affected survival. In fact, although a recent metanalysis by Smith et al. showed that HIV infection decreases the rate of spontaneous clearance by about 10%, especially in IDUs, they also showed that the spontaneous clearance rate decreases with age [21]. In our cohort, co-infected PLWH were more likely to be younger, and the younger age might have acted as a confounder, masking the real effect of HCV infection. 

In our study, male sex and younger age are associated with a more favorable prognosis in terms of long-term survival. 

Nowadays, early diagnosis of the infection is the key to successful management of HIV, allowing for prompt initiation of an antiretroviral regimen and lowering the risk of disease progression [13]. The progressive reduction in mortality rate and improvements in life expectancy are the result of appropriate initiation of antiretrovirals before PLWH present with advanced HIV disease and of the availability of more potent drugs with less side effects [22].

The goal of modern ART is to obtain persistent undetectability of HIV-RNA plasma viral load (“virological success”) and immune reconstitution (“immunological success”). Persons initiating ART at very low CD4+ cell counts are at higher risk of opportunistic infections and death [23]. These PLWH are unlikely to achieve a meaningful immune recovery [23]. Moreover, late presenters have higher rates of morbidity and mortality [23].

Our findings support the results obtained by Ren et al. [24]. They showed that not only has the baseline CD4+ cell count been proven to be an important predictor for the long-term outcome of ART and PLWH survival, as previously reported in the literature, but also the dynamics of CD4+ cell count across the follow-up period could be very important [25].

Many studies have established a clear association between lower total CD4+ cell counts and the development of opportunistic infections in HIV subjects [26,27]. The incidence increases significantly when the CD4+ lymphocytes count falls to less than 200/µL [28]. CD4+ lymphocytes play an important role in the immune system; they promote B cell antibody production and release cytokines in response to specific antigenic stimulation [28]. The INSIGHT START study is the first large-scale randomized clinical trial that provided concrete scientific evidence on the benefit of starting the antiretroviral treatment in all asymptomatic HIV-infected individuals, regardless of CD4+ cell count [29]. 

Our study has some limitations that should be addressed. Firstly, this is an observational, retrospective analysis. Furthermore, it is a single-center study, and it consists of a limited sample size. Finally, some data about clinical and laboratory status of PLWH were missing in the medical records. A possible bias in our analysis is that not all the subjects began treatment at the same time after diagnosis; moreover, they were diagnosed at vastly different stages of infection, and almost none of them ever had any testing before diagnosis. In addition, we do not have any data about adherence to treatment. Many of them were already dead by the time HAART was available. However, it also has some strengths. First of all, the data collection was performed in a single center in which the medical staff have operated since 1985, at the beginning of the HIV pandemic in our area, with an exciting blend of memories, dusty paper archives, and rudimentary electronic files. Moreover, the retention in care of such a high percentage of subjects (93.7%) in the same clinical center for such a long time has been crucial for a complete data collection.

## 5. Conclusions

This retrospective observational study showed that 44% of PLWH diagnosed during the first decade of the HIV pandemic survived more than 25 years from their first diagnosis. This was an unimaginable result at the beginning of the pandemic, and to share this information could considerably push the screening campaign and the access to care for unaware or hesitant people. Moreover, it confirmed that conditions traditionally associated with late presentation, such as older age and lack of ARV treatment, are associated with bad prognosis. This study also demonstrates declining death rates among HIV-infected persons in our cohort with access to HAART. The availability and accessibility of effective antiretroviral therapy for people living with HIV remain the cornerstone of survival. Prompt initiation of ART and adherence to the treatment remain the stronger predictors of outcome. Therefore, early diagnosis and linkage to care of PLWH play a key role in the HIV care continuum and represent the necessary first steps to antiretroviral therapy initiation and viral suppression, with the purpose of reducing the risk of developing HIV transmission, HIV-related complications, and death.

## Figures and Tables

**Table 1:** Clinical and epidemiological characteristics of the whole cohort.

Characteristics	Population (n = 210)	N	%
Sex	M *	158	75.24%
	F *	52	24.76%
Age (median, IQR *)	28 (25–34) years		
Year of HIV * diagnosis	1983–1989	95	45.24%
	1990–1994	115	54.76%
HCV * positivity	Yes	101	48.10%
	No	100	47.62%
	Not known	9	4.29%
HBV * positivity	Yes	5	2.38%
	No	196	93.33%
	Not known	9	4.29%
Risk factors	IDUs *	100	47.62%
	MSMs *	42	20%
	Heterosexual transmission	50	23.81%
	Multiple transfusions	13	6.19%
	Not known	5	2.38%
AIDS * presenter	Yes	69	32.86%
	No	139	66.19%
	Not known	2	0.95%
AIDS * during the period of interest	Yes	116	55.24%
	No	92	43.81%
	Not known	2	0.95%
CD4^+^ at diagnosis, cell/µL, median, IQR	234 (IQR 54–439)		
CD4^+^ nadir at diagnosis, cell/µL, median, IQR	35 (IQR 14–94)		

* M—male; F—female; IQR—interquartile range; HIV—human immunodeficiency virus; HCV—hepatitis C virus; HBV—hepatitis B virus; IDU—injection drug user; MSMs—men who have sex with men; AIDS—acquired immunodeficiency syndrome.

**Table 2:** Long-term survivors and causes of death.

		N	%
LTS *		93	44.29
NLTS *		116	55.24
Not known		1	0.48
Cause of death	AIDS *-related	98	83.05
	HCV *-related	9	7.63
	Non-AIDS * malignancy	4	3.39
	Cardiovascular diseases	4	3.39
	Overdose	3	2.54

* LTS—long-term survivor; NLTS—non-long-term survivor; AIDS—acquired immunodeficiency syndrome; HCV—hepatitis C virus.

**Table 3:** Year of diagnosis and AIDS at diagnosis.

		Year of Diagnosis										
		1983	1985	1986	1987	1988	1989	1990	1991	1992	1993	1994
No AIDS *	NLTS *	0	10	4	6	4	3	8	5	7	1	1
	LTS *	1	17	8	8	6	2	11	9	11	8	9
AIDS *	NLTS *	1	3	4	2	5	10	7	14	13	6	1
	LTS *	0	0	0	0	0	0	0	0	1	0	2

* AIDS—acquired immunodeficiency syndrome; NLTS—non-long-term survivor; LTS—long-term survivor.

## Data Availability

The data presented in this study are available on request from the corresponding author.

## Supplementary Materials

The following supporting information can be downloaded at: https://www.mdpi.com/article/10.3390/idr15010008/s1. Figure S1: Median time of survival in PLWH who had access to HAART, stratified per CD4^+^ T-cell count at diagnosis.
