# Supplementary figures and images for: Long-Term Survivors in a Cohort of People Living with HIV Diagnosed between 1985 and 1994: Predictive Factors Associated with More Than 25 Years of Survival
Source: Infect Dis Rep. 2023 Jan 20;15(1):70–83. doi: 10.3390/idr15010008 (PMC9957088; doi:10.3390/idr15010008)

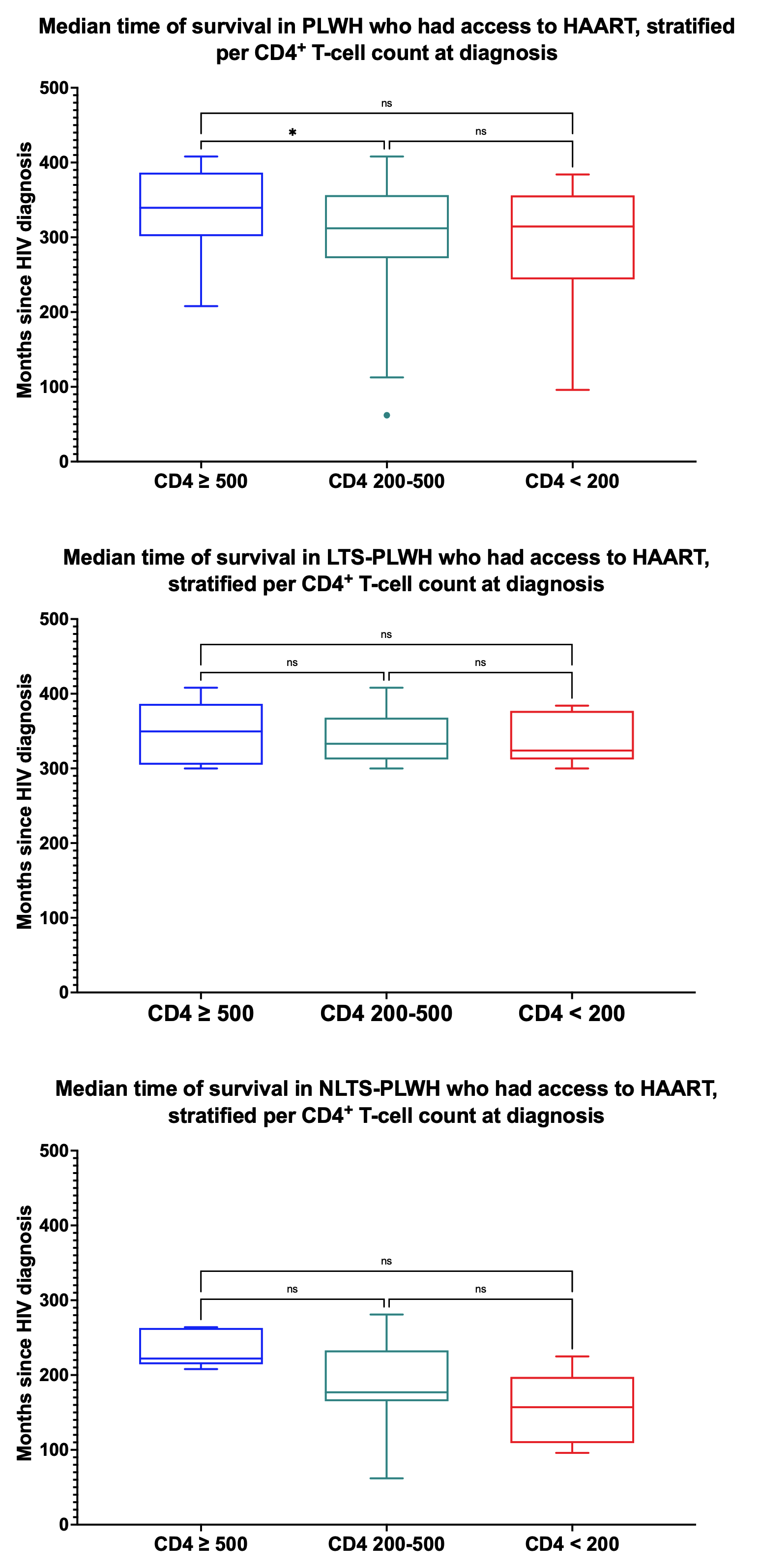

Supplement: Supplementary file 1 [file idr-15-00008-s001.zip › idr-2030648-supplementary.tiff]
